# Larval hematopoietic organs of multiple *Drosophila* species show effector caspase activity and DNA damage response

**DOI:** 10.17912/micropub.biology.001392

**Published:** 2024-12-18

**Authors:** Deepak Maurya, Bama Charan Mondal

**Affiliations:** 1 Cytogenetics Laboratory, Department of Zoology, Banaras Hindu University, Varanasi, Uttar Pradesh, India

## Abstract

Macrophages are present in various forms throughout metazoans and play conserved roles in phagocytosis, immunity, and tissue homeostasis. In *Drosophila melanogaster'* s larval hematopoietic organ, the lymph gland, transient caspase-mediated activation of caspase-activated DNase triggers the DNA damage response (DDR), which is crucial for macrophage-type cell differentiation. Here, we report that other *Drosophila* species having different-sized mature lymph glands show effector caspase activity and DDR similar to those in *Drosophila melanogaster* , indicating that the developmental mechanism regulating phagocytic macrophage differentiation is conserved in different species of *Drosophila* .

**Figure 1. Active caspase and DNA damage found in the lymph glands of multiple Drosophila species:**
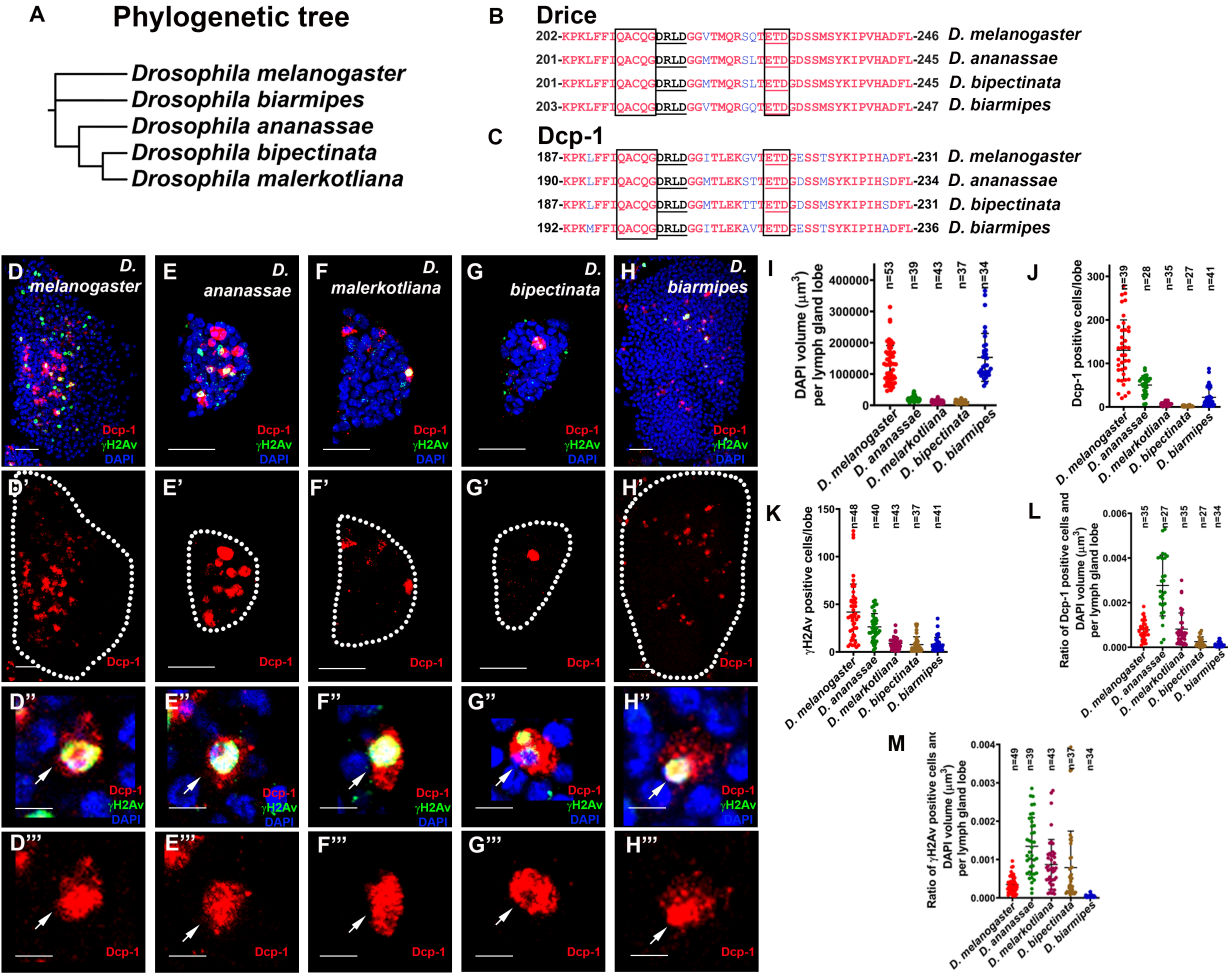
**(A)** The schematic diagram represents the phylogeny of five *Drosophila* species: *D. melanogaster* , *D* . *ananassae* , *D* . *malerkotliana* , *D* . *bipectinata,* and *D. biarmipes.* **(B-C)** Multiple sequence alignment of the c-terminal region of Drice and Dcp-1 in *D. melanogaster* , *D* . *ananassae* , *D* . *bipectinata,* and *D. biarmipes.* The red highlighted area shows identical sequences, the cleavage tripeptide region (ETD), the active pentapeptide region shown in a rectangular box, and the alternative cleaved site displayed in black underline (DRLD). **(D-D''')** The lymph gland of *D. melanogaster* shows Dcp-1 and γH2Av co-staining with DAPI ( **D** ) and only Dcp-1 staining ( **D'** ). The high-magnification image (arrow) shows co-staining of Dcp-1 and γH2Av with DAPI ( **D''** ) and only Dcp-1 staining ( **D'''** ). **(E-E''')** The lymph gland of *D. ananassae* shows Dcp-1 and γH2Av co-staining with DAPI ( **E** ) and only Dcp-1 staining ( **E'** ). The high-magnification image (arrow) shows the co-staining of Dcp-1 and γH2Av with DAPI ( **E''** ) and only Dcp-1 staining ( **E'''** ). **(F-F''')** The lymph gland of *D. malerkotliana* shows Dcp-1 and γH2Av co-staining with DAPI ( **F** ) and only Dcp-1 staining ( **F'** ). The high-magnification image (arrow) shows the co-staining of Dcp-1 and γH2Av with DAPI ( **F''** ) and only Dcp-1 staining ( **F'''** ). **(G-G''')** The lymph gland of *D. bipectinata* shows Dcp-1 and γH2Av co-staining with DAPI ( **G** ) and only Dcp-1 staining ( **G'** ). The high-magnification image (arrow) shows the co-staining of Dcp-1 and γH2Av with DAPI ( **G''** ) and only Dcp-1 staining ( **G'''** ). **(H-H''')** The lymph gland of *D. biarmipes* shows Dcp-1 and γH2Av co-staining with DAPI ( **H** ) and only Dcp-1 staining ( **H'** ). The high-magnification image (arrow) shows the co-staining of Dcp-1 and γH2Av with DAPI ( **H''** ) and only Dcp-1 staining ( **H'''** ). **(I)** Quantification of DAPI volume per lymph gland lobe in all five species. **(J)** Quantification of Dcp-1-positive cells per lymph gland lobe in all five species. **(K)** Quantification of γH2Av-positive cells per lymph gland lobe in all five species. **(L)** Quantification of the ratio of Dcp-1 positive cells and DAPI volume per lymph gland lobe in all five species. **(M)** Quantification of the ratio of *γ* H2Av positive cells and DAPI volume per lymph gland lobe in all five species. All images are shown from the late third instar larval lymph gland lobe. The whole lymph gland lobe images are the maximum intensity projections of the middle third optical section image with a scale bar of 25 μm. All high-magnification images are single optical sections with a scale bar of 5 μm. The staining of Dcp-1 is shown in red, γH2Av in green, and DAPI in blue. All images represent three or more independent biological experiments, and n represents the number of lymph gland lobes.

## Description

Macrophages that destroy foreign substances by engulfing them were discovered in 1882 by Élie Metchnikoff in starfish larvae. He described the process as phagocytosis (Underhill et al., 2016) . Subsequent investigations have demonstrated that macrophages are conserved throughout metazoans, exhibiting additional functions in regulating development, tissue repair, homeostasis, and innate immunity (Lazarov et al., 2023; Park et al., 2022) . In triploblastic animals, phagocytic cells travel through the coelomic cavity because of an open circulatory system and remove cellular debris or pathogens (Maheshwari, 2022; Banerjee et al., 2019) . In mammals, resident tissue macrophages develop from the yolk sac and erythro-myeloid precursors during an early embryonic stage and have self-renewal capacity throughout life. Monocyte-derived macrophages are also associated with rapidly replenishing tissues, such as in the intestine (Lazarov et al., 2023; Lee & Ginhoux, 2022; Park et al., 2022) . During the evolution from single-celled organisms to highly complex vertebrates, the roles of macrophages and the phagocytic process have remained largely conserved (Yutin et al., 2009) . However, the mechanism underlying phagocytic macrophage differentiation remains unclear.

*Drosophila* *melanogaster* provides an excellent genetic model for studying macrophage development and innate immunity. It has only myeloid-type cells comprising 95% plasmatocytes, with functional similarity to mammalian macrophages; 5% crystal cells act similar to mammalian platelets; and lamellocytes are large flat cells seen only after infection or injury. As in mammals, hematopoiesis in *Drosophila* *melanogaster* occurs in multiple waves. The first wave starts from the embryonic head mesoderm and produces macrophages and crystal cells that persist into the larval and adult stages. The second wave begins at the late embryonic stage in the hematopoietic tissue, called the lymph gland. The lymph gland matures by the late third instar larval stage, and it disintegrates during metamorphosis, contributing to adult macrophages (Banerjee et al., 2019; Shim, 2015) .

Caspases are generally involved in programmed cell death. However, studies have suggested that they also play non-lethal roles in cellular differentiation and development (Burgon & Megeney, 2018; Larsen & Sørensen, 2017). Recently, we reported that caspase-mediated activation of caspase-activated DNase (CAD) causes DNA strand breaks in the differentiating progenitors of the lymph gland, which are required for macrophage differentiation (Maurya et al., 2024) . Here, we extend this finding and show that caspase-mediated DNA damage is necessary for lymph gland development in other *Drosophila* species phylogenetically close to *D. melanogaster* (Schawaroch, 2002; Singh, 2016) , viz., *D. ananassae* , *D* . *malerkotliana,* *D. bipectinata* , and *D. biarmipes* ( **Figure 1A** ).

To check the effector caspase activity, we performed multiple sequence alignments of Death related ICE-like caspase (Drice) and Death caspase-1 (Dcp-1) protein sequences in these species, except for *D* . *malerkotliana,* whose genome sequence was not available in the NCBI database. We detected highly conserved sequences with more than 80% identity in the two effector caspases, Drice and Dcp-1 (highlighted in red in **Figures 1B–1C** ) in the C-terminal region, which included the cleavage site tripeptide ETD region (black box, underlined), alternative cleavage site DRLD (underlined), and active site pentapeptide QACQG (black box) (Song et al., 1997) .

To examine the presence of lymph glands in these species, we dissected the late third instar larvae (just before they became white pupae). As in *D. melanogaster,* all the four species had lymph glands at the anterior region of the dorsal side, although their sizes differed. *D. ananassae, D* . *malerkotliana* , and *D. bipectinata* had smaller lymph glands, whereas *D. biarmipes* had the same size as *D. melanogaster* ( **Figures 1D–1H** , quantification in **Figure 1I** ). It is the first report demonstrating the presence of lymph glands in these *Drosophila* species.

The effector caspases Dcp-1 and Drice are activated after cleavage by the initiator caspase Dronc, which generates the active large subunit that can be detected by an anti-Dcp-1 antibody (Cat # 9578S, Cell Signaling Technology), which binds only to the cleaved large subunit of both effector caspases (Li et al., 2019) . Therefore, to check for caspase-mediated DNA damage during lymph gland development, we co-immunostained the late third instar larval lymph glands of the four species with anti-Dcp-1 and anti-γH2Av (a marker of DNA damage response). Remarkably, the lymph glands of all the species studied exhibited effector caspase activity and DNA damage ( **Figures 1D–1H'** ). High-magnification images confirmed that caspase activity (Dcp-1 staining) and DNA damage (γH2Av staining) were present in the same cells ( **Figures 1D''–1H'''** ). However, the number of Dcp-1 and γH2Av positive cells in the three species having smaller lymph glands (measured by DAPI volume) was lesser than in *D* . *melanogaster* ( **Figures 1J–1K** ) *.* A comparison of the ratio of Dcp-1-positive cells with the DAPI volume of the lymph gland revealed that caspase activity was higher in *D. ananassae* , equal in *D. malerkotliana* , and low in *D. bipectinata* and *D. biarmipes* ( **Figure 1L** ). The ratio of γH2Av-positive cells with DAPI volume in *D* . *ananassae* , *D* . *malerkotliana,* and *D* . *bipectinata* was higher than that in *D* . *melanogaster* but lower in *D. biarmipes* ( **Figure 1M** ) *.* These results suggest that caspase activation and DNA damage response in the lymph glands were highly conserved among the species studied.

Caspase-mediated programmed cell death (apoptosis) has evolved as an evolutionarily conserved mechanism to eliminate unwanted cells. However, caspases also play various non-apoptotic roles in development, cellular differentiation, and survival in invertebrates and vertebrates (Fujita et al., 2008; Maurya et al., 2024; Solier et al., 2017) , including monocyte-to-macrophage differentiation in mammals (Niu et al., 2017; Solier et al., 2023) . In *Drosophila melanogaster* , caspase-activated DNase causes DNA strand breaks that regulate macrophage differentiation (Maurya et al., 2024) . This study showed that the cellular mechanism regulating macrophage differentiation is evolutionarily conserved in *Drosophila* . Studies have revealed that DNA strand breaks cause changes in the chromatin landscape, triggering heterogeneous gene activation (Dehingia et al., 2022; Larsen et al., 2022; Puc et al., 2017) . A detailed study is required to determine how DNA strand breaks regulate the chromatin landscape and gene expression during the development of evolutionarily conserved phagocytic macrophages.

## Methods

**Fly stocks used in the study**

We used *Drosophila melanogaster, Drosophila ananassae, Drosophila bipectinata, Drosophila malerkotliana,* and *Drosophila biarmipes* , all of which were available in our departmental stock collection (Banerjee & Singh, 2017) . All flies were raised on standard fly food (Maurya et al., 2024) in an incubator (PHCBI Model #MIR-554-PE) maintained at 25°C.

***Drosophila* larval lymph gland dissection and immunohistochemistry**

Lymph glands of the wandering third instar larvae were dissected in chilled 1X PBS (phosphate-buffered saline) and fixed in 4% paraformaldehyde (Thermo Fisher Scientific, Cat# 28908) for 30 min. The tissues were then washed thrice with 0.3% PBST (0.3% Triton X-100 in 1X PBS) for 15 min each. After incubation in blocking solution (0.1% Triton X-100, 0.1% BSA, 10% FCS, 0.1% sodium deoxycholate, and 0.02% Thiomersal, in 1XPBS) for 30 min, they were incubated overnight in the desired primary antibody at 4°C, following which the tissues were washed three times with 0.3% PBST and incubated in a blocking solution for 30 min. Finally, the tissues were incubated with a secondary antibody at room temperature for 2 h, washed in 0.3% PBST three times, counterstained with DAPI (4',6-Diamidino-2-Phenylindole, Dihydrochloride, Thermo Fisher Scientific, Cat# D1306), and mounted in DABCO (1,4-diazabicyclo [2.2.2] octane, Sigma, Cat# D27802, 2.5% DABCO in 70% glycerol made in 1X PBS) (Maurya et al., 2024) .

Primary antibodies used for immunohistochemistry were rabbit anti-Dcp1 (1:100, Cell Signaling Technology, Cat # 9578S) and mouse anti-γH2Av (1:1000, DSHB, UNC93-5.2.1-s). The secondary antibodies (1:200 dilutions) were donkey anti-rabbit Alexa Fluor 555 (Invitrogen), goat anti-rabbit Alexa Fluor 647 (Invitrogen), goat anti-mouse Alexa Fluor 488 (Jackson ImmunoResearch), and goat anti-mouse Cy3 (Jackson ImmunoResearch).

**Microscopy, image processing, and analysis**

Images were acquired on a Zeiss LSM-900 confocal microscope using Zen software (version 3.4) with a 40X objective and identical imaging settings for all tissues. ImageJ software (NIH, USA) was used for image processing. Adobe Photoshop software was used to assemble the figure panel. The maximum intensity projection of the middle third stack of the lymph gland was used to better represent the inside of the lymph gland. For clarity, the white dotted line in Figure 1 demarcates lymph gland boundaries. At least three independent biological replicates and three experimental replicates were analyzed. The Interactive Tree of Life (iTOL) version 7.0 (https://itol.embl.de) was used to construct the phylogenetic tree, and NCBI BLAST was used to align the sequences.

**Quantification of lymph gland phenotypes**

All quantifications were performed using ImageJ software (NIH, USA). The number of γH2Av and Dcp-1 positive cells was counted manually in both lobes of the primary lymph gland and analyzed separately. The procedure to determine the threshold was performed during image processing to select pixels of interest based on the intensity of the pixel values, following which the ‘‘Measure stack'' plugin was used to find each optical section's fluorescent area in DAPI staining. The fluorescent area in each optical section was added and multiplied by the stack interval (2 μm) to determine the volume (Yang et al., 2019) . Quantification graphs were prepared using GraphPad Prism 9 software.
